# COVID-19 and financial market response in China: Micro evidence and possible mechanisms

**DOI:** 10.1371/journal.pone.0256879

**Published:** 2021-09-09

**Authors:** Zhan Wang, Zhongwen Zhang, Qiong Zhang, Jieying Gao, Weinan Lin

**Affiliations:** 1 School of Finance, Capital University of Economics and Business, Beijing, China; 2 School of Economics and Management, Tsinghua University, Beijing, China; 3 School of Public Administration and Policy, Renmin University of China, Beijing, China; 4 College of Economics & Management, South China Agricultural University, Guangzhou, China; University of Almeria, SPAIN

## Abstract

This paper uses event study based on the Generalized Autoregressive Conditional Heteroscedasticity (GARCH) model to study the impact of the COVID-19 outbreak on China’s financial market. It finds that the pandemic had an overall significant and negative impact on the stock prices of firms listed on SSE, SZSE and ChiNext. However, such impact appeared to be heterogeneous across industries, affecting listed firms in industries such as pharmaceutical and telecommunications positively, but those in services industries such as accommodation, catering, and commercial services negatively. Apparently, a crisis for some had been an opportunity for others. In addition, this paper seeks to understand the micro mechanism behind the heterogeneity of pandemic shock from the perspective of firms’ financial position. It finds that listed firms with higher debt level were hit harder, whereas those with more net cash flow had displayed higher resilience against the blow of the pandemic. However, the opposite pattern is found among those listed on ChiNext and in industries severely devastated by the pandemic. These findings have policy implications in terms of preventing systemic financial risks and facilitating recovery during pandemic-induced economic downturns. It also helps investor adjust investment strategies, hedge against risks, and secure gains when the market conditions in general are unfavorable.

## 1. Introduction

The black swan of COVID-19 has dealt a huge blow to the global economy and brought turmoil to financial markets worldwide. By the end of July, globally confirmed COVID-19 cases from more than 200 economies had surpassed 200 million, with over 4 million fatalities. In a bid to slow the spread of the coronavirus, many countries and regions imposed nationwide or citywide lockdowns. However, such measures have a huge economic cost—economic activities have been halted throughout the world, sending the GDP growth of major economies into negative territory in the first half of this year. Disrupted supply chains, anemic demands and pandemic-induced panic have also wreaked havoc on the global capital market, with the majority of assets, excluding a handful of safe-havens such as gold and grains [1, 2], experiencing price drops to various degrees and increasingly volatile price swings. Meanwhile, multiple circuit breakers have been triggered in stock and futures markets in US, UK, Germany and France, among other countries. The magnitude and speed of the fall in asset prices and the range of assets suffering extreme price declines can only find parallels during the 1930 Great Depression and the 2008 global financial crisis [3].

The COVID-19 outbreak first came to the notice of China’s public health departments and media attention in early January. On January 20, Zhong Nanshan, head of the high-level expert team under China’s National Health Commission, confirmed human-to-human transmission of the coronavirus and cases of infection among medical workers. Immediately after, the Chinese government took stringent control measures, closed the city of Wuhan on January 23 and expanded the lockdown to other cities and towns in Hubei province to prevent the spread of the disease. By March, domestic outbreak was largely put under control. This outbreak was a public health crisis unprecedented in terms of speed, scale and the challenges that it posed to the national emergency response system since the founding of New China. Its economic repercussion is severe: GDP in the first quarter fell by 6.8% year on year, recording negative growth in the first time since 1992 when China started to report quarterly GDP. Outside China, COVID-19 has quickly travelled across the world; though domestic outbreaks have gradually come under control in some countries and regions, when this pandemic would end remains uncertain to date. The systemic impact of the COVID-19 pandemic as a major global public health crisis on economies and financial markets is a topic that has been widely discussed. Many scholars have explored it from multiple angles [4].

Significant external shocks tend to have striking impacts on macroeconomic and financial indicators. Over the last decades, economic, financial, political, environmental, and health crises have spurred financial researchers to quantify their impacts on stock market returns. The COVID-19 is considered to be a “once-in-a-century pathogen” [4]. Given China’s status as the second largest economy and the high degree of coupling between global financial markets [5], a systemic and quantitative assessment of the pandemic-induced shock to China’s financial market will enrich relevant research and provide valuable reference for other countries and regions. This paper performs event study based on the Generalized Autoregressive Conditional Heteroskedasticity (GARCH) model and choose daily transaction and quarterly financial statement data of companies listed on Shanghai Stock Exchange (SSE), Shenzhen Stock Exchange (SZSE) and ChiNext to quantify the overall and heterogeneous impact of the pandemic on China’s financial market and explore the underlying mechanism from the point of view of corporate financial position. The results show that the COVID-19 pandemic has dealt a negative blow to listed firms market-wide and across major sectors. At a more disaggregated level, sizable heterogeneity exists among different industries: the pandemic has decimated accommodation, catering and commercial services, but brought a big boom for pharmaceutical and telecommunication industries, suggesting that crises can always generate opportunities. In addition, from a corporate finance point of view, this paper finds that firms with higher debt levels tend to suffer more from the pandemic, whereas those with larger net cash flows are less likely to be negatively affected by the pandemic. When investigating whether China’s startup board and the main boards reacted differently to the pandemic shock, this paper finds that, as compared with well-established firms trading on the main boards, firms listed on ChiNext are typically small- and medium-sized innovative firms with higher debt-to-asset ratios, which make them more vulnerable to the negative impact of the pandemic. In fact, for innovative firms, due to the newness of their business models and fragile operations, those previously recording good cash flow performance have reacted more severely to the shock. This paper further looks at firm performance in sectors more severely hit by the pandemic such as the service sector, and finds that all firms experienced a massive, homogeneous shock from the pandemic and were confronted with broad-based operational challenges, even complete shutdowns. Firms in a better cash position before the crisis saw more fluctuations in their cash flow performance, and their share prices thus suffered more damage under the shock of the pandemic.

This paper makes the following contributions. First, it incorporates GARCH into event study to more accurately assess the impact of COVID-19 on China’s stock market because as Brockett *et al*. [6] point out, the classical event study methodology without accounting for GARCH effects could lead to inappropriate conclusions. Second, given the high degree of correlation between global financial markets and the stronger role of financial contagion in generating stock return volatility during the COVID-19 period [7], it further analyzes the uneven effects of COVID-19 across industries in SSE, SZSE and ChiNext. The results obtained at the sectoral level point to the benefit of portfolio diversification, since although all markets suffered a crash during the same periods, it is still possible to find sectors to reduce investment risk. This paper adds to the body of research measuring the impact of COVID-19 on financial market and financial contagion. More importantly, compared with the handful of studies looking at how the pandemic affects the financial market in China at sectoral level [8], this paper unveils at micro level how firms’ financial position affects their ability to withstand the pandemic shock from the perspectives of firm liabilities and cash flow management. It finds that listed firms with higher debt level were hit harder, whereas those with more net cash flow had displayed higher resilience against the blow of the pandemic. However, the opposite pattern is found among firms listed on ChiNext and in industries severely devastated by the pandemic. These empirical findings have policy implications in terms of preventing systemic financial risks and supporting growth during economic downturns caused by crises similar to the pandemic, and can help guide investor actions in hedging against risks and gaining returns despite unfavorable environment.

The rest of the paper is structured as follows. Section 2 is a review of exiting literature. Section 3 describes model specification and data choice, introducing the source of data and the construction of variables after a brief explanation of event study based on the GARCH model. In Section 4, estimates of the pandemic’s impact on the entire financial market and across major sectors are presented first, followed by its heterogeneous effect at sub-sector level. Section 5 explores the mechanism behind the uneven impact of the pandemic across industries and firm types from a financial position point of view. The last section concludes.

## 2. Literature review

As an on-going global public health crisis, the COVID-19 pandemic has brought both supply and demand shocks to the world economy. As of the end of July, the global spread of the virus has yet to be effectively contained, and the ensuing economic chaos and financial market turmoil persist. Centering on the impact of pandemics on economies and financial markets, scholars at home and abroad have already carried out quite a number of studies.

First, the pandemic has caused a significant shock at the macroeconomic level. Ludvigson *et al*. [9] estimate that COVID-19 could lead to a fall of 12.75% in industrial production, 17% loss in service sector employment, sustained reductions in air traffic, and heightened macroeconomic uncertainty for up to five months. According to Mulligan [10], shutdowns in the US during the pandemic has reduced market production by 25–28% in the short run, incurring an economic loss of $7 trillion and an employment loss of 28 million. Baker *et al*. [11] suggest that the pandemic has substantially increased uncertainty in the economy, likely causing a year-on-year contraction of 11% in real GDP in the US, half of which can be attributed to pandemic-induced uncertainty. Based on UnionPay daily transaction data at city level, Chen *et al*. [12] find that offline consumption has slumped by 42% as a result of the pandemic, of which products and services consumption dropped by 44% and 43%, respectively. When further disaggregated, catering and entertainment consumption plunged by 72% and tourism by 64%. China is estimated to have suffered a loss in offline consumption totaling over 1 trillion yuan, equaling 1% of GDP in 2019, within two months after the outbreak. Baker *et al*. [13] point out changes in the consumption habits of American households as the pandemic crisis gets worse: initial quick rise in consumption expenditure, mainly in retail, credit card spending and food items, was followed by a sharp decline in overall spending; this pattern was most prominent in states imposing shelter-in-place orders; moreover, social distancing measures were the main reason behind the decrease in restaurant and retail spending. Based on a survey of over 5800 small businesses, Bartik *et al*. [14] find that the pandemic has led to large-scale temporary closures and layoffs, with the number of employee counts down by 40% relative to January; many businesses have also suffered financial distresses—the median business with monthly expenses over $10,000 has less than one month of cash on hand; moreover, 43% of businesses have suspended their operations. In terms of policy effect, the measures implemented did not completely meet firm needs. Coibion *et al*. [15] find that employment loss caused by COVID-19 was significantly larger than officially reported: the number of jobs lost is estimated to reach 20 million, far greater than the number over the entire Great Recession; what’s worse, many of those losing jobs were not actively looking for new ones, and labor force participation fell by 7 percentage points during the period of investigation, outstripping the 3 percentage point decline that occurred cumulatively over 2008–2016. Also looking at the labor market in the US, Forsythe *et al*. [16] find that nearly all industries and occupations (excluding essential retail and nursing) saw contraction in postings and spikes in unemployment insurance initial claims. The authors further point out that the broad-based deterioration of the labor market is more closely related to the spread of the virus itself than to stay-at-home policies. Research from a gender perspective by Alon *et al*. [17] further show that the employment drop related to social distancing measures had a large impact on sectors with high shares of female employment. On the other hand, Aum *et al*. [18], find that low-skilled workers and the self-employed suffered the most from the pandemic and also from government policies forcing people to work from home.

Second, the impact of the pandemic on various industries has also drawn considerable research attention. Wu *et al*. [19] find that at industry level, the COVID-19 pandemic has the greatest short-term impact on consumer- and labor-intensive industries in China. For example, the output value of the service industry fell 6.3% compared to normal. Fu and Shen [20] find that the pandemic has had a significant and negative effect on the performance of energy companies. When goodwill impairment was introduced as a moderating variable, companies with goodwill impairment were more strongly affected by the pandemic. Focusing on China’s insurance market, Wang *et al*. [21] find that income from commercial insurance premium, monthly year-on-year growth rate of premium, insurance density, and insurance depth have all decreased due to COVID-19. The negative impacts on property and personal insurances are both statistically significant. And the adverse impact of the pandemic on the insurance market can be alleviated by raising the level of social security and digital insurance. Using input-output analysis, Duan *et al*. [22] find that China’s response measures and suppressed demand elasticity can limit the long-term impact of the pandemic on the economy, but in the short term, service industries such as transportation, tourism and entertainment could decline by as much as 18%. Gunay and Kurtulmus [23] investigate the impact of social distancing on the US service sector. Their findings show that the pandemic initially affected mainly the entertainment and airline industries, with gradual deterioration in the hotel industry, led by small-market-cap companies. However, the authors find no evidence of a negative impact on the restaurant industry from the pandemic in their analysis period. Gunay *et al*. [24] investigate the impact of the first wave of the COVID-19 pandemic on various sectors of the Australian stock market, as well as the financial contagion between the Chinese stock market and Australian Stock market. Results show high time-varying correlations between the Chinese stock market and most of the Australian sector indices, with the financial, health care, information technology, and utility sectors displaying a decrease in co-movements during the pandemic. When the firm size is considered, smaller companies in the energy sector exhibited gradual deterioration, whereas small firms in the consumer staples sector experienced the largest positive impact from the pandemic.

Furthermore, a vast number of studies find that financial market did not escape the shock caused by the pandemic. Alfaro *et al*. [25] predict the change in the number of COVID-19 infections for any given trading day based on the changes in infection cases in the previous two trading days, and estimates that a doubling of projected infections corresponds to a decrease in market value of 4 to 11%. The authors have also shown that fluctuations in the market become less volatile when the trajectory is more predictable. In the study by Baker *et al*. [26], the authors use text-based methods to look for potential explanations for major stock market jumps since 1900. They find that no previous infectious disease outbreak, including the Spanish Flu, could parallel the shock on the US stock market from the COVID-19 pandemic. The reason for this enormous stock market reaction, much more so than to previous pandemics in 1918–19, 1957–58 and 1968, can be attributed to government restrictions on commercial activities and voluntary social distancing, which caused huge damage to the US economy dominated today by service industries. Focusing on stock market reactions in China, Yang *et al*. [27] note that under the shock of the COVID-19, China’s A share market opened on February 3 with a nosedive after being closed during the Spring Festival holiday: as panicked investors rushed to sell, nearly 3000 shares listed on Shanghai and Shenzhen stock exchanges plunged to their limit-down level. Liu and Wang [28] discusse irrational factors that characterize the outbreaks of infectious disease, and under- and over-reactions to such extreme events. Distinguishing between the direct impact of outbreaks and the indirect effects resulting from irrational factors, the authors provide ideas for future research on epidemics from an economic or financial point of view and suggestions for possible policy responses. Fahlenbrach et al. [29] document the impact of firms’ financial flexibility in the face of pandemic-induced revenue shortfalls on their stock returns, pointing out that less financially flexible firms had significantly lower returns on their stocks and saw smaller rebound in their stock prices after the government announced the lifting of lockdowns. Ramelli *et al*. [30] find that firms more exposed to trade with China underperformed in the US stock market at the initial stage of the COVID-19 outbreak. However, as the virus spread to Europe and the US, corporate debt and cash holdings emerged as important value drivers, relevant even after the Fed intervened in the bond market. The phenomenon of high market correlations and financial contagion during different type of crises has also attracted many scholars’ attention. Kenett *et al*. [31] find that the high degree of coupling between global financial markets has made the financial village prone to systemic collapses. Kenett et al. [32] also find that while the developed “western” markets (US, UK, Germany) are highly correlated, the interdependencies between these markets and the developing “eastern” markets (India and China) are volatile and with noticeable maxima at times of global world events. Vidal-Tomás et al. [33] propose an early warning indicator based on the collective movement of stock prices in a given market, investors can reduce the risk of their portfolio while policy-makers can set more efficient policies to avoid the effects of financial instability on the real economy. Ali et al. [34] investigate the reaction of financial markets globally in terms of their decline and volatility as coronavirus epicentre moved from China to Europe and then to the US. Their findings suggest that the earlier epicentre China has stabilized while the global markets have gone into a freefall especially in the later phase of the spread. Even the relatively safer commodities have suffered as the pandemic moves into the US. Grilli et al. [35] review the literature on credit market models by emphasizing the mechanisms able to generate financial crises and contagion.

In sum, existing research typically explores the impact of the COVID-19 pandemic at macroeconomic level, focusing on a specific country or region. Studies quantifying its shock on China’s financial market are few and far between, let alone ones that look at the heterogeneous effects across industries and firm types and seeks to provide an explanation. Therefore, this paper will enrich and complement previous works in relevant areas by adopting GARCH-based event study to analyze daily transaction and quarterly financial statement data of firms listed on SSE, SZSE and ChiNext.

## 3. Model specification and data selection

An event study is a method frequently seen in financial studies to measure the impact of an event, which is adopted in this paper to assess the “abnormal change” in the target variables caused by an external shock—the COVID-19 pandemic—over event window, and quantify the effect of the pandemic on firms and industries in China. Similar to the Difference in Difference (DID) design widely used in studies evaluating policy effects at micro level, event studies seek to capture an event’s “extra” or “marginal” effect on target variables mainly by comparing the difference between what has been observed in the real world and the behavior of a control group, which provides a baseline for when everything else is unchanged. Compared with DID, event studies offer a better tool for charting and understanding the event’s effect on target variables over the entire window period. This method was first proposed by Dolley [36] to analyze how common stock split-ups affected stock returns. Many scholars after him, including Dyckman et al. [37] and Cowan [38] perfected it. It has found its use in a broad range of fields including economics, finance, management and social sciences.

This paper bases its analysis on the theory of event studies and daily transaction data of listed firms, which are sourced from the WIND database. The sample of listed firms covers all firms trading on the main boards of SSE and SZSE and ChiNext, a total of 3439 firms after excluding IPO firms and firms experiencing financial distress (i.e., “special treatment” firms designated with the prefix “ST” in front of their stock codes). In event study method, there are three main models for calculating abnormal returns: the average adjusted return rate model; the market index adjusted return rate model; and the market model [39]. The average adjusted rate of return model has a large deviation, when a bull or bear market occurs on the event day [40]. The market index adjusted return model has a strong relationship assumption, which is not applicable in most cases [39]. Market models are the most commonly used and have good predictive power [41]. In this article, we used the market model to calculate abnormal returns (AR).

Given that the market model obtained based on daily transaction data may not satisfy normal distribution and may show a volatility clustering tendency, we correct it with the GARCH (1,1) approach used in the study by Chen et al. [42] rather than relying on the traditional OLS estimation for the event study. In this process, only 2772 firms can achieve convergence and satisfy the conditions for correction, which form our final sample for later analysis.

Specifically, the abnormal return *AR* for the stock of listed firm *i* on day *t* can be calculated using the following formula:

$${AR}_{i,t}=R_{i,t}-E(R_{i,t})=\varepsilon_{i,t},\;\varepsilon_{i,t}|\varphi_{i,t-1}∼(0,h_{i,t})$$ (1)

Where $E(R_{i,t})={\hat{\alpha }}_{i}+{\hat{\beta }}_{i}R_{m,t}$ is the expected “normal return” for firm *i* as reflected by the market model, in which *R*_*i*,*t*_ and *R*_*m*,*t*_ represent the yield rate for firm *i* on day *t and* the index yield rate of the market which the firm *i* is listed on, and can be calculated through *R*_*i*,*t*_ = *log*(*P*_*i*,*t*_/*P*_*i*,*t*−1_) and *R*_*m*,*t*_ = log(*P*_*m*,*t*_/*P*_*m*,*t*−1_) (*P*_*i*,*t*_ and *P*_*i*,*t*−1_ are the closing prices for firm *i* on day *t* and *t-1*, respectively; *P*_*m*,*t*_ and *P*_*m*,*t*−1_ are the respective closing prices for SSE Composite Index, SZSE Component Index or ChiNext Index on day *t* and *t-1*, depending on whichever the stock exchange firm *i* is listed on; ${\hat{\alpha }}_{i}$ and ${\hat{\beta }}_{i}$ are the corresponding maximum likelihood estimators; *ε*_*i*,*t*_ is the error term; *φ*_*i*,*t*−1_ represents all the known information on day *t-1*).

The conditional variance *h*_*i*,*t*_ under the GARCH (1, 1) assumption can be expressed as:

$$h_{i,t}=\omega_{i}+\delta_{i}h_{i,t-1}+\gamma_{i}\varepsilon_{i,t-1}^{2},\;of\;\mathrm{which},\;\omega_{i}>0,\;\gamma_{i}>0,\;\delta_{i}>0,\;\gamma_{i}+\delta_{i}<1$$ (2)

In an event study, the precise identification of event day, event window and estimation window is of the utmost importance. Since the lockdown of Wuhan was announced at 2 o’clock in the morning on January 23, 2020, and on the same day, the whole city entered a war-like state, with other regions across China raising emergency response levels, we consider January 23 as the event day (day 0). This choice is consistent with existing research looking at COVID-19’s impact on the Chinese economy [12]. Event window is a period of time over which the event exerts its influence. Given that the event may have already captured attention before it happens and its impact may linger on for a while after the event, the event window often consists of a number of days before and after the event date. Estimation window is a time period more or less comparable to the event window period but not exposed to the event’s effect, which can be used to observe the “normal” (counterfactual) behavior of target variables without the influence of the event. On this account, we define an event window of 41 trading days from December 25, 2019 to February 28, 2020, including the day of the event (day 0) and 20 trading days before and after the event (-20, +20). The estimation window is set to cover 150 trading days prior to the event (-170, -21) from May 10 to December 24, 2019.

After obtaining the parameters for the market model ${\hat{\alpha }}_{i}$ and ${\hat{\beta }}_{i}$ based on the daily transaction data during the estimation window, we enter them into Eq (1) to estimate the abnormal returns of stock *i* on day *t* during the event window. The cumulative abnormal returns (CAR) for the period T_1_−T_2_ can be written as:

$${CAR}_{i,\;(T_{1},T_{2})}=\sum_{T_{1}}^{T_{2}}{AR}_{i,t}$$ (3)

For assessing the heterogeneous impact of this public health event on different industries in later section, we also specify the equation for the average abnormal return (cross-sectional abnormal return, AAR) on day *t* for the whole sample and industry-specific subsamples, as well as the average cumulative abnormal return as of day *t* (cross-sectional cumulative abnormal return, CAAR) as follows (in which N refers to the size of the whole sample and that of the industry-specific subsamples, respectively):

$${AAR}_{t}=\frac{\sum_{i=1}^{N}{AR}_{i,t}}{N},\;{CAAR}_{T_{1},T_{2}}=\sum_{j=1}^{N}\frac{{CAR}_{{j,(T}_{1},T_{2)}}}{N}$$ (4)

## 4. Quantifying the COVID-19 pandemic’s impact

This section first analyzes the overall impact of the COVID-19 pandemic on China’s publicly traded firms, and then moves on to look more closely at industry-specific reactions to this shock event.

### 4.1 Overall impact on the stock market

**Fig 1:**
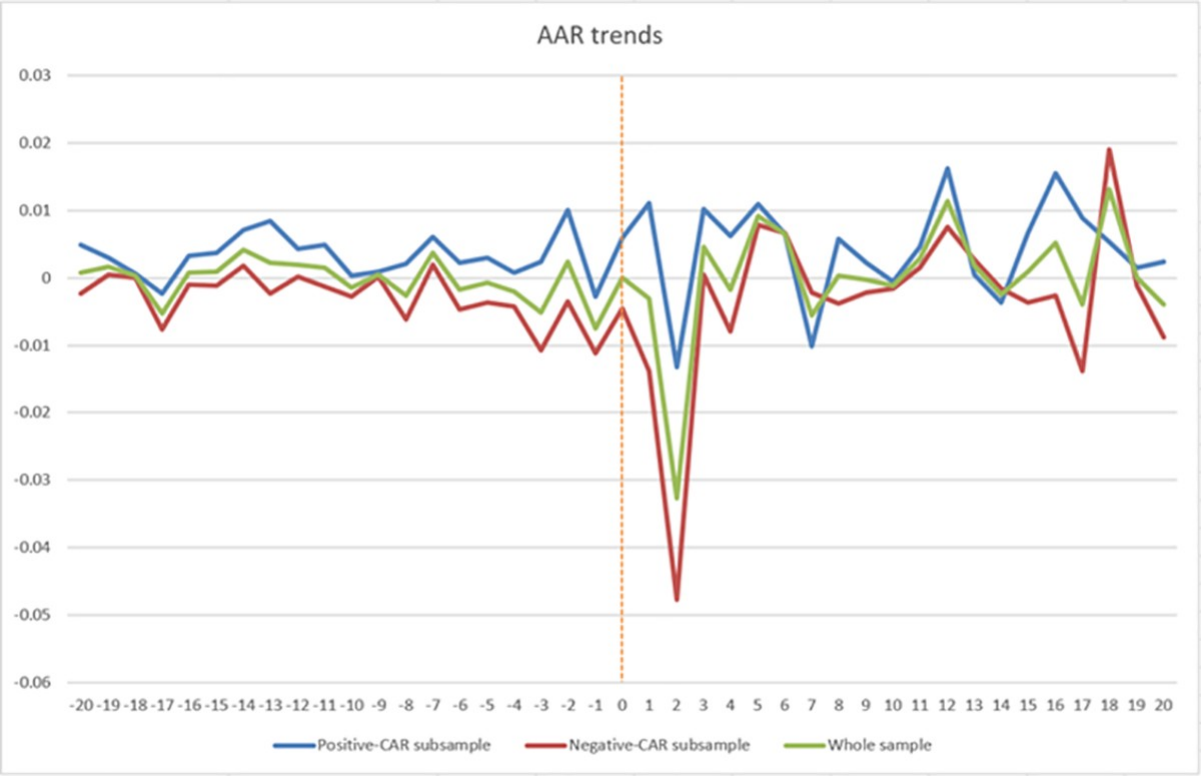
AAR trends over the 41-day event window (-20, +20). Note: The dashed red vertical line indicates the event day (January 23, 2020), and the horizontal axis shows trading days within the event window.

Fig 1 portraits the pattern of ARs over the 41-day event window (-20, +20) for the whole sample. Clearly, the COVID-19 outbreak marked by the lockdown of Wuhan dealt a huge short-term blow to the whole-sample AAR (the drop in AAR is extremely sharp in the first (day +1) and second day (day +2) after the event), and the fluctuations in AAR become visibly more volatile for a period after the event day (day 0). Prior to event day, the virus had already been circulating in communities in Wuhan and the ongoing investigations by the Chinese Center for Disease Control and Prevention (briefly as China CDC) had also been drawing broad media attention, to which the whole-sample AAR reacted negatively. The reactions became more notable starting from day -3 (i.e., January 20, three days before the event day when Zhong Nanshan confirmed human-to-human transmission and the infection of medical staff). We also divide the whole sample into two subsamples based on whether a firm’s CAR during the event window (-20, +20) is positive or negative (1135 and 1637 listed firms, respectively) and calculate the AAR for each subsample. For the subsample with positive CAR, its AAR was positive on the event day and day +1, but turned negative on day +2. The subsample with negative CAR reported negative AAR on the event day, day +1 and day +2, with the fall in AARs being particularly pronounced for the latter two days. Overall, AARs for the whole sample, the positive-CAR subsample, and the negative-CAR subsample all experienced more volatility after the event day. However, the pandemic’s negative shock on the whole sample of listed firms primarily came from the subsample with negative CAR.

### 4.2 Industry-specific impact: Broad grouping

To further investigate the impact of COVID-19 on different industries, we estimate industry-specific AARs during the event window period at 1-digit level (broad grouping). The results are given in Table 1.

**Table 1:** Industry-specific AARs over the event window.

Event window Industry	-20	-15	-10	-5	-4	-3	-2	-1	0	1	2	3	4	5	10	15	20
Agriculture, forestry, animal husbandry and fishery	-0.50***	0.62*	0.17	-0.37	-0.39	-1.25**	-1.1***	-1.57***	-1.25***	-3.04***	-4.05***	-1.71***	2.22***	-0.32	-0.06	-0.69***	-2.05***
Mining	-0.32	-0.30**	-2.41***	-0.10	0.05	-0.32	-0.25	-1.07***	-1.01***	-2.56***	-2.34***	0.25	-0.45**	-0.14	0.14	0.63**	-1.44***
Manufacturing	0.07	-3.13***	-0.22***	-0.03	-0.26***	-0.29***	0.47***	-0.76***	0.06	-0.17	-3.13***	0.59***	-0.33***	0.73***	-0.09	0.17***	-0.52***
Production and supply of electricity, heat, gas and water	-0.31***	-0.36***	-0.26**	-0.42***	-0.25*	-0.65***	0.26*	-0.53***	0.10	-2.29***	-2.47***	0.04	-0.39**	0.78***	-0.37**	-0.42***	0.02
Construction	-0.27	-0.10	0.05	-0.04	0.10	-0.53**	-0.09	-0.52***	0.18	-1.65***	-4.58***	-0.09	-0.27	0.66**	-0.25**	-0.20	0.03
Wholesale and retail trades	0.07	0.14	-0.03	-0.07	-0.22	-0.10**	0.53**	-1.2***	-0.18	0.44*	-2.61***	0.74***	0.22	0.09	-0.55***	0.03	0.39
Transport, storage and post	-0.13	-0.28**	0.28*	-0.01	-0.11	-0.39	0.00	-0.61***	0.31*	-1.12***	-3.49***	0.40*	-0.18	-0.39**	-0.33**	-0.22	0.20
Accommodation and catering	-0.06	-1.02	-0.39	-0.26	-0.34	-1.82	-1.01	-0.11	0.02	-1.88	-7.30***	-0.30	-0.93**	3.68***	-0.54	-2.74***	-1.06
Information transmission, software and information technology	0.83***	1.11***	0.40**	-0.21	-0.05	-1.59***	-0.83***	-0.60***	0.21	1.06***	-4.43***	-0.08	0.35*	3.89***	-0.34**	1.27***	-0.67***
Finance	-0.00	-0.01***	-0.00**	-0.00**	0.00*	-0.00	0.00	0.00	-0.00	0.01	-0.03***	-0.00***	-0.00***	-0.00	0.01***	-0.01***	-0.00**
Real estate	-0.00	-0.00	0.00**	0.00	0.00	-0.01**	-0.00	-0.00	-0.00**	-0.02***	-0.04***	0.00	-0.01***	0.00	0.01***	-0.00*	0.01**
Leasing and commercial services	0.19	1.10**	0.06	0.22	-0.40	-2.30***	-1.30***	-0.63*	-0.25	-0.88*	-5.22***	0.18	-0.64**	1.08**	0.10	-0.63*	-0.74**
Scientific research and technical services	0.05	-0.54*	0.36	-0.27	-0.27	-0.05	0.91**	-0.79**	0.42	-0.73	-3.20***	0.20	-0.81*	0.83	-0.36	-0.72***	1.11*
Management of water conservancy, environment and public facilities	-0.13	-0.38***	-0.09	-0.45**	-0.29**	-1.16***	-0.56**	-1.21***	-0.24	-1.92***	-4.75***	0.45	1.20**	4.60***	-0.77***	-0.98***	0.00
Education	0.15	-0.54	0.76	-0.51	-0.37	-1.50	-0.10	-1.89	-1.04	-2.23	2.29	2.93*	3.39**	-1.93*	-2.39*	0.33	0.18
Health and social services	0.08	-1.26**	1.12	1.24	-0.50	0.26	1.82*	-4.16**	-1.80	-0.40	1.72	1.26	1.41	-1.09	-0.93**	-0.76*	1.09
Culture, sports and entertainment	0.36	1.80***	-0.72***	-0.2	-0.55	-3.09***	0.22	-1.64***	-0.39	0.06	-2.86***	1.58***	1.67***	1.63***	-1.75***	-0.96***	-0.52

Note

***, ** and *, indicate significance at the 1%, 5% and 10% level, respectively.

Here, considering the second day after the event day (day +2) was the most severely hit, the results in Table 1 are ranked by industry-specific AARs on day +2. As we can see, the majority of 1-digit industries all reported negative and statistically significant AARs on the event day, day +1, and day +2. Only a handful of industries such as health and social services had positive but insignificant AARs. Clearly, the COVID-19 bludgeoned industries on a broad base, sending share prices down sharply. Focusing on the period from January 20 (day -3) to day +5 can better show the short-term impact of the pandemic on firms’ stock performance. From Table 1, we can find 10 1-digit industries negatively impacted by the pandemic for 4 days and longer, namely, “agriculture, forestry, animal husbandry and fishery”, “mining”, “manufacturing”, “production and supply of electricity, heat, gas and water”, “construction”, “transport, storage and post”, “information transmission, software and information technology”, “real estate”, “leasing and commercial services”, and “management of water conservancy, environment and public facilities”, corresponding to 2387 firms, 86.11% of the 2772 firms in the whole sample. This further illustrates the extensive short-term impact of the COVID-19 pandemic across industries and firms.

Next, we analyze stock market’s short-term reaction to the pandemic based on industry-specific CAARs within the 9-day event window (-3, +5). The corresponding results are listed in Table 2. As it shows, almost all industries recorded negative CAARs. After the event day, all 17 industries had negative CAARs, 13 industries had negative and significant CAARs, and 2, namely, “leasing and commercial services” and “accommodation and catering”, registered drops in CAARs beyond -9%, suggesting firms from these industries took a grave hit from the pandemic.

**Table 2:** Industry-specific CAARs at 1-digit level over the 9-day event window.

Event window Industry	-3	-2	-1	0	1	2	3	4	5
Leasing and commercial services	-2.30***	-3.60***	-4.24***	-4.49***	-5.37***	-10.58***	-10.40***	-11.04***	-9.96***
Accommodation and catering	-1.82	-2.83	-2.94	-2.93	-4.80**	-12.10***	-12.40***	-13.34***	-9.66***
Agriculture, forestry, animal husbandry and fishery	-1.25**	-2.36***	-3.92***	-5.18***	-8.22***	-12.26***	-10.55***	-8.33***	-8.66***
Mining	-0.32	-0.57	-1.64***	-2.66***	-5.22***	-7.56***	-7.31***	-7.76***	-7.9***
Real estate	-0.53**	-0.67*	-0.83**	-1.30***	-2.81***	-6.50***	-6.50***	-7.20***	-7.03***
Construction	-0.53**	-0.62*	-1.14***	-0.96**	-2.61***	-7.19***	-7.28***	-7.55***	-6.89***
Transport, storage and post	-0.39	-0.39	-1.00**	-0.70	-1.82***	-5.30***	-4.90***	-5.08***	-5.47***
Production and supply of electricity, heat, gas and water	-0.65***	-0.39**	-0.92***	-0.82***	-3.11***	-5.59***	-5.55***	-5.94***	-5.16***
Finance	-0.02	0.04	0.08	-0.13	0.40	-2.36***	-3.24***	-3.82***	-3.86***
Management of water conservancy, environment and public facilities	-1.16***	-1.72***	-2.93***	-3.17***	-5.10***	-9.85***	-9.40***	-8.20***	-3.60**
Scientific research and technical services	-0.05	0.86	0.06	0.49	-0.24	-3.43**	-3.23**	-4.05**	-3.22*
Culture, sports and entertainment	-3.09***	-2.87***	-4.51***	-4.90***	-4.84***	-7.70***	-6.12***	-4.45**	-2.83
Manufacturing	-0.29***	0.18	-0.58***	-0.52***	-0.69***	-3.81***	-3.22***	-3.55***	-2.82***
Wholesale and retail trades	-0.10	0.43	-0.77*	-0.94	-0.50	-3.11**	-2.37*	-2.15	-2.06
Information transmission, software and information technology	-1.59***	-2.42***	-3.02***	-2.82***	-1.75***	-6.19***	-6.27***	-5.92***	-2.03**
Health and social services	0.26	2.08	-2.08	-3.87	-4.28**	-2.56	-1.29	0.12	-0.98
Education	-1.50	-1.59	-3.48	-4.52	-6.75	-4.46	-1.53	1.86	-0.07

Note

***, ** and *, indicate significance at the 1%, 5% and 10% level, respectively.

### 4.3 Industry-specific impact: Narrow grouping

Since a broad grouping at 1-digit level may not effectively reveal the uneven effect of the COVID-19 pandemic on different industries and firms, we further disaggregate the impact at 2-digit level under two classification schemes, one drafted by the state statistical bureau (titled Industrial Classification for National Economic Activities, ICNEA for short hereafter) and the other adopted by the China Securities Regulatory Commission (CSRC). Table 3 presents CAARs over the 9-day event window under the ICNEA scheme. We see negative pandemic shock for most of the industries, of which, the 5 worst affected industries were “wood processing and manufacture of wood, bamboo, rattan, palm, and straw-made articles”, “catering”, “public facility management”, “ancillary mining activities” and “architectural decoration and other construction”. On the other hand, industries such as “manufacture of medical products” and “postal services” had positive and significant CAARs as high as 13.67% and 19.17%, respectively.

**Table 3:** Industry-specific impact at 2-digit level under ICNEA scheme, 9-day event window.

Industry	CAAR	Industry	CAAR	Industry	CAAR
Wood processing and manufacture of wood, bamboo, rattan, palm, and straw-made articles	-12.71***	Manufacture of general-purpose machinery	-7.15***	Manufacture of paper and paper products	-3.06
Catering	-12.68***	Railway transport	-7.08**	Software and information technology services	-3.01**
Public facility management	-11.02***	Real estate	-7.03***	Manufacture of chemical raw materials and chemical products	-2.85***
Ancillary mining activities	-10.82***	Storage	-6.91	Capital markets services	-2.69*
Architectural decoration and other construction	-10.45***	Air transport	-6.74***	Manufacture of computers, communication and other electronic equipment	-2.53***
Commercial services	-10.30***	Forestry	-6.58**	Agricultural services	-2.19***
Manufacture of furniture	-9.98***	Renovation	-6.30***	Processing of petroleum, coking, processing of nuclear fuel	-1.46
Agriculture	-9.75***	Mining and washing of coal	-6.22***	Health care	-0.98
Construction of buildings	-9.49	Metal products manufacturing	-5.98***	Telecommunications, radio, television, and satellite transmission services	-0.61
Production and supply of gas	-9.08***	Road transport	-5.91***	Ecological protection and environmental management	-0.39
Manufacture of liquor, soft drinks and refined tea	-9.05***	Production and distribution of electric power and heat power	-5.80***	Education	-0.07
Manufacture of leather, fur, feather and related products and footwear	-9.04***	Manufacture of electrical machinery and equipment	-5.75***	Food manufacturing	0.53
Manufacture of textile and apparel	-9.00***	Manufacture of rubber and plastics	-5.63***	Printing and recorded media	0.89
Radio, television, film and television recording and production	-8.88***	Building projects	-5.59***	Wholesale trade	0.98
Other manufacturing	-8.69**	Manufacture of Automobiles	-5.18***	Internet and related services	1.11
Animal husbandry	-8.67**	Manufacture of railway, ships, aerospace and other transportation equipment	-5.13**	Production and distribution of tap water	2.19
Nonferrous metals mining and dressing	-8.54***	Processing of food from agricultural products	-4.85***	Manufacture of articles for culture, education, art, sports, and entertainment	3.42
Culture and arts	-8.37	Loading/unloading, removal, and other transport services	-4.69**	Research and experimental development	4.34
Manufacture of measuring instruments	-8.31***	Retail trade	-4.54***	Manufacture of chemical fibers	5.19
Smelting and pressing of nonferrous metals	-8.28***	Smelting and processing of ferrous metals	-4.53**	Conglomerates	5.46
Accommodation	-8.14**	Polytechnic services	-4.40**	News and publishing	6.32*
Extraction of petroleum and natural gas	-7.94**	Insurance	-4.24*	Manufacture of textiles	6.64
Fishery	-7.65***	Monetary and financial services	-3.81***	Comprehensive use of waste resources	10.92**
Nonmetal mineral products	-7.31***	Manufacture of special purpose machinery	-3.64***	Manufacture of medicines	13.67***
Other financial activities	-7.28***	Ferrous metals mining and dressing	-3.50	Postal services	19.17**
Water transport	-7.15***	Leasing	-3.33		

Note

***, ** and *, indicate significance at the 1%, 5% and 10% level, respectively.

Table 4 displays CAARs over the 9-day event window under the CSRC scheme. Similar to the results in Table 3, the majority of the industries were negatively affected by the pandemic. The 5 hardest hit industries were “agriculture”, “tourism”, “light manufacturing”, “mining”, and “retail”, reporting CAARs of -17.67%、-15.32%、-14.84%、-13.08%、-13.03%, respectively. Meanwhile, significant and positive CAARs were recorded in “biological products”, “traditional Chinese medicine Ⅱ”, “chemical-pharmaceutical”, “medical equipment”, “healthcare product distribution”, and “telecommunication operations”. These 6 industries managed to generate CAARs of 11.4%、13.13%、13.47%、14.36%、20.49%、46.41%, respectively.

**Table 4:** Industry-specific impact at 2-digit level under CSRC scheme, 9-day event window.

Industry	CAAR	Industry	CAAR	Industry	CAAR
Agriculture conglomerates	-17.67***	High and low voltage equipment	-7.50***	Rubber	-3.67
Tourism conglomerates	-15.32***	Other construction materials	-7.23***	Motor Ⅱ	-3.66
Other light manufacturing	-14.84***	Electronic equipment manufacturing	-7.14***	Papermaking Ⅱ	-3.00
Mining services	-13.08***	Cement manufacturing	-7.14***	Other leisure	-2.76
Specialty retail	-13.03***	Railway transport	-7.08**	其Other mining	-2.57
Catering Ⅱ	-12.68***	Real estate development	-6.96***	Computer applications	-2.46**
Renovation and decoration	-12.10***	Auto assembly	-6.91***	Packaging printing	-2.03
Ship manufacturing	-11.13***	Apparel and textile	-6.72***	Securities Ⅱ	-1.78
Tourist attractions	-10.52***	Special-purpose Equipment manufacturing	-6.68***	Food processing	-1.76
Measuring instrument manufacturing	-10.27***	Coal mining	-6.60***	Computer equipment	-1.67
Livestock and poultry breeding	-10.11***	Forestry Ⅱ	-6.58**	Culture and media	-1.47
Gold Ⅱ	-9.41**	Steel Ⅱ	-6.54***	Environmental engineering	-0.66
Other transport	-9.30***	Building construction	-6.50**	Electronic components Ⅱ	-0.22
Beverage manufacturing	-9.15***	Aero equipment	-6.45**	Animal health	0.06
Air transport	-8.91***	Hotel Ⅱ	-6.27**	Industrial park development	0.46
Landscaping	-8.91***	Electricity	-5.88***	Agriculture product processing	0.53
Industrial metals	-8.86***	Infrastructure construction	-5.82***	Water utilities Ⅱ	1.08
Gas Ⅱ	-8.83***	Petroleum extraction	-5.67***	Medical services	1.45
Commercial property	-8.83***	Air transport Ⅱ	-5.63	Petrochemicals	2.05
Precious metals	-8.78***	Public transport Ⅱ	-5.61**	Conglomerates Ⅱ	2.31
Metal products	-8.67**	Power supply equipment	-5.61***	Internet media industry	2.89
Electric automation	-8.47***	Plastics	-5.16	Chemical raw materials	4.02
Marketing communication	-8.44***	Auto parts	-5.05***	Other electronics	4.57
Auto services	-8.42***	Telecommunications equipment	-5.03***	AV equipment	4.87
Farming	-8.11***	Highway	-5.01***	Aerospace equipment	4.97
Metals and nonmetals	-7.91***	Military equipment for land applications	-4.58	Semiconductor	5.11*
Ports Ⅱ	-7.84***	Chemical products	-4.30***	Chemical fiber	5.19
light manufacturing for home use	-7.79***	Insurance Ⅱ	-4.24*	Textile manufacturing	7.43
Optics and optoelectronics	-7.73***	White goods	-4.2**	Biological products	11.4**
Fodder Ⅱ	-7.68***	Airports Ⅱ	-4.02	Traditional Chinese medicine Ⅱ	13.13***
Fishery	-7.65***	Logistics Ⅱ	-3.96*	Chemical-pharmaceutical	13.47***
Trade Ⅱ	-7.64***	Professional engineering	-3.91**	Medical equipment	14.36***
Non-traditional finance	-7.62***	Glass manufacturing	-3.86	Healthcare product distribution	20.49***
General machinery	-7.53***	Banking Ⅱ	-3.81***	Telecommunication operations	46.41***
General retail	-7.52***	Transport equipment	-3.75		

Note

***, ** and *, indicate significance at the 1%, 5% and 10% level, respectively.

As Tables 3 and 4 demonstrate, while the impact of COVID-19 on the financial market was wide-ranging and overall negative, its sectoral distribution was uneven. The pandemic affected supply and demand through consumer and firm behaviors, for example, demand for certain industries plunged because people’s heightened self-protection awareness led to reduced economic activities. On the production side, fear of infection would hamper labor participation and delay work resumption [43]. Research on SARS by Liu Taoxiong and Peng Zongchao [44] suggests that non-essential economic activities featuring high human density or frequent interaction with the public bear the brunt of epidemics: culture, sports, leisure, tourism and catering businesses are often among the first to see a fall in demand after an outbreak; demand for contact-intensive but less avoidable activities like transportation may shrink in accordance to the severity of the epidemics; on contrast, essential activities requiring zero or infrequent face-to-face interaction may not suffer much, or even increase during the outbreaks. Our analysis also shows that the pandemic hurt contact-intensive, non-necessity industries most, including tourism, retail and catering. Nevertheless, as in every crisis lies opportunities, during the COVID-19 pandemic, massive demand for masks, antiviral drugs and other related products drove the production and investment in the healthcare sector, generating significant and positive CARs in “healthcare product distribution”, “medical equipment”, “traditional Chinese medicine”, “chemical-pharmaceutical”, and “biological product” industries. At the same time, fueled by the pandemic, online shopping and smart economy experienced an explosive growth. New business models and services such as fresh produce e-commerce, telemedicine, online education and remote working quickly emerged and expanded, boosting stock market performance of the telecommunication operations and courier services sectors.

## 5. Micro mechanism behind the pandemic’s heterogeneous impact

The previous section has shown that the share price reactions of listed firms to the COVID-19 pandemic were overwhelmingly negative, with a few exceptions. In several industries, firms actually benefited from the outbreak and earned positive “abnormal” returns. In this section, we seek to understand the micro mechanism behind the heterogeneity of pandemic shock from the perspective of firms’ financial position.

### 5.1 Research hypothesis

Financial states affect firms’ stock price, and different capital structure could lead to different response to external shocks [45]. Firms have to repay their debts as scheduled, which means that an unexpected external shock when the debts are due could trigger bankruptcy risk. During the COVID-19 outbreak, many firms were forced to halt operations and became cash-strapped. Among them, those with a higher debt-to-asset ratio faced significantly higher bankruptcy risk. In line with these observations, we propose the first research hypothesis as follows:

*Hypothesis 1*: *Firms with higher debt level suffer more due to the pandemic*.

The pandemic caused huge demand loss, and the resultant main operational risk was cash flow disruptions caused by temporary market shutdown. Some firms require strong and sustained cash flow to fund their daily operations. Once their cash flows are disrupted or completely halted, these firms may find themselves unable to carry on normal activities and thereby become less attractive to investors [46]. Firms with high net cash flow typically demonstrate stronger financial resilience and risk resistance. When an external shock hits, they can draw on their cash reserves to cover shortfalls in cash flow and maintain operations. A such, we postulate the second hypothesis:

*Hypothesis 2*: *Firms with higher net cash flow prior to the pandemic are more resistant to the negative shock of the pandemic*.

### 5.2 Variable definition and empirical model constructing

To test Hypothesis 1 and 2, we choose the CARs during the 9-day event window (*CAR*_*i*_) as the dependent variable to reflect the extent to which a firm was impacted by the pandemic. Debt-to-asset ratios reported in firms’ 2019 annual reports are used to reflect their debt level (Lev_i_) [47]. Also from the 2019 annual reports, we source net cash flow data for the variable net cash flow (Cash_i_) [48–52] and treat the data in the following way: for any given firm, we make a sum of its net cash flow, the absolute value of the minimum net cash flow in the sample, and the constant 1, and then calculate the natural logarithm of the sum to ensure the monotonic transformation of non-negative net cash flow values. By definition, net cash flow refers to the difference between a firm’s cash inflows and outflows resulting from its economic activities (including operating, investing, financing activities and non-recurring items) in a certain accounting period. We also select from literature a number of major control variables that affect firms’ share prices. For example, the study by Lustig and Leinbach [53] on companies with a small market capitalization shows that firm size (*Size*_*i*_, proxied by market capitalization, or market cap) affects share prices and abnormal returns, Bodie *et al*. [54] point out that under the efficient market hypothesis, rate of return on total assets (*ROA*_*i*_) and Tobin’s Q ratio (*TobinQ*_*i*_) are important variables in asset pricing. Basu [55] suggests the existence of a positive correlation between the change in share prices and price-earnings ratio (*PE*_*i*_) when the market is efficient. Based on the observation of pandemic-induced shutdowns across the world, we can safely assume that net inventory (*Inventory*_*i*_) also has a significant impact on the operation of listed firms and their stock investors. As such, we eventually choose firm size (*Size*_*i*_), price-earnings ratio (*PE*_*i*_), net inventory (*Inventory*_*i*_), rate of return on total assets (*ROA*_*i*_) and Tobin’s Q ratio (*TobinQ*_*i*_) as our control variables [56–59]. For the first three control variables, we use natural logarithm of data collected from firms’ 2019 annual report. Finally, we employ a dummy to account for industry fixed effects. Data for independent variables and control variables are all sourced from the CSMAR database. The regression function for H1 and H2 can be expressed as below:

$${{CAR}_{i}=\alpha_{0}+\alpha_{1}{Lev}_{i}+\alpha_{2}{Cash}_{i}+\alpha_{3}{Size}_{i}{+\alpha }_{4}{TobinQ}_{i}+\alpha_{5}{PE}_{i}+\alpha_{6}Inventory}_{i}+\alpha_{7}{ROA}_{i}+Industry+\varepsilon_{i}$$ (5)

A summary of the descriptive statistics of the variables is given in Table 5. For the whole sample, CAR has a mean of -3.04% and a standard deviation of 14.08%, which indicates substantial cross-sectional variation in CAR across firms. The mean of CAR is -9.85% in the negative-CAR subsample, and 12.95% in the positive-CAR subsample. The debt-to-asset ratio has a mean of 41% for the whole sample and 42% and 39% for the positive- and negative-CAR subsample, respectively. The mean of net cash flow after treatment is 26.42, 26.41, and 26.44 for the whole sample, the negative-CAR subsample, and the positive-CAR subsample, respectively.

**Table 5:** Descriptive statistics of all variables.

Variable	N	mean	sd	min	p50	max	mean (Positive-CAR subsample)	mean (Negative-CAR subsample)
CAR (%)	2288	-2.89	13.38	-31.22	-5.37	95.54	-9.23	12.62
Lev	2288	0.41	0.19	0.01	0.4	0.97	0.42	0.39
Cash	2288	26.42	0.56	0.04	26.44	26.79	26.41	26.44
Size	2288	22.86	1.21	20.67	22.64	28.22	22.84	22.92
PE	2288	3.57	1.01	0.12	3.46	8.57	3.57	3.56
Inventory	2288	19.87	1.97	7.20	19.84	27.52	19.86	19.88
ROA	2288	0.05	0.05	0.00	0.04	0.53	0.05	0.06
TobinQ	2288	1.83	1.26	0.69	1.46	15.49	1.78	1.96

### 5.3 Empirical analysis and findings

Table 6 presents the corresponding regression results. The first part of the table lists the results from the whole sample regression, of which, Columns 1–3 summarize results for when only debt-to-asset ratio, only cash flow, and both are considered, respectively. Clearly, debt-to-asset ratio has a significantly negative and cash flow a significantly positive impact on CAR. On average, an increase of 1 standard deviation (0.19) in debt-to-asset ratio is associated with a decrease of 0.82 percentage points in CAR, whereas an increase of 1 standard deviation (0.56) in the natural logarithm of cash flow corresponds to 0.21 percentage point increase in CAR.

**Table 6:** Baseline regression results.

	Whole sample			Negative-CAR subsample			Positive-CAR subsample		
	(1)	(2)	(3)	(4)	(5)	(6)	(7)	(8)	(9)
Lev	-0.0434**		-0.0442**	-0.0230**		-0.0233**	-0.0009		0.0001
	(-2.23)		(-2.27)	(-2.27)		(-2.30)	(-0.02)		(0.00)
Cash		0.0039***	0.0041***		0.0018***	0.0019***		0.1055	0.1055
		(3.16)	(3.21)		(3.40)	(3.47)		(1.09)	(1.09)
Size	0.0130***	0.0113***	0.0133***	0.0090***	0.0081***	0.0091***	-0.0089	-0.0081	-0.0081
	(3.96)	(3.51)	(4.01)	(5.06)	(4.72)	(5.10)	(-1.25)	(-1.15)	(-1.12)
PE	-0.0100**	-0.0091**	-0.0099**	-0.0091***	-0.0086***	-0.0090***	-0.0041	-0.0041	-0.0041
	(-2.43)	(-2.22)	(-2.41)	(-4.16)	(-3.99)	(-4.14)	(-0.50)	(-0.51)	(-0.51)
Inventory	0.0006	-0.0002	0.0007	-0.0004	-0.0008	-0.0004	0.0021	0.0021	0.0021
	(0.32)	(-0.08)	(0.37)	(-0.39)	(-0.74)	(-0.35)	(0.43)	(0.41)	(0.41)
ROA	-0.1417	-0.0737	-0.1428	-0.1830***	-0.1469***	-0.1828***	-0.2734	-0.2747	-0.2746
	(-1.58)	(-0.86)	(-1.59)	(-3.64)	(-3.09)	(-3.62)	(-1.55)	(-1.62)	(-1.55)
TobinQ	0.0012	0.0014	0.0011	-0.0022	-0.0021	-0.0022	0.0035	0.0034	0.0034
	(0.40)	(0.49)	(0.40)	(-1.34)	(-1.29)	(-1.34)	(0.58)	(0.56)	(0.56)
Industry fixed effect	Y	Y	Y	Y	Y	Y	Y	Y	Y
Obs.	2288	2288	2288	1624	1624	1624	664	664	664

Notes

1) The t values are shown in brackets

2) all results reported in the table above are net of industry fixed effect at 1-digit level

3) ***, ** and *, indicate significance at the 1%, 5% and 10% level, respectively.

Next, we divide the sample into two subsamples of negative or positive CARs and present their corresponding results in the second part (Columns 4–6) and third part (Columns 7–9) of the table. As shown, in the negative-CAR subsample, the estimated coefficients for debt-to-asset ratio and cash flow are both significant and exhibit negative and positive signs, respectively. However, neither reach statistical significance in the positive-CAR subsample, suggesting that the negative impact of debt-to-asset ratio and the positive impact of cash flow on CAR can be mainly attribute to the reactions of firms negatively affected by the pandemic. Taken together, results in Table 6 provide evidence that a higher proportion of debt in the capital structure of listed firms make them more vulnerable to stock market crashes. This finding is consistent with conclusions from existing research [60, 61]. Meanwhile, firms light on debt showed relatively strong risk resistance during this black swan event. Moreover, this outbreak severely disturbed, or even halted firms’ cash flow due to temporary economic shutdowns. Those with larger net cash flow before the crisis hit were more resilient to the shock and performed better in terms of share prices during the outbreak.

As for controlled variables, market cap had a positive effect for the whole sample and the negative-CAR subsample, implying that firms with larger market cap suffered less pandemic-induced damage. Market cap is often used to measure the size of publicly trading firms, and larger firms are more capable of withstanding risks. During the pandemic, large-cap firms had more resources at their disposal. They were also able to absorb unexpected liquidity fluctuations in the financial market and reduce stock turnover due to swings in market sentiment [56, 62]. That is why they were less influenced by the pandemic. In the whole sample and the negative-CAR subsample, price-earnings ratio had a significant and negative impact, meaning that firms with higher price-earnings ratios were more susceptible to the negative shock of the pandemic. Financial market price changes constantly with the change in market sentiment and economic cycle, and firms’ stock premium could fall sharply under the influence of negative external factors [58]. As such, firms that have higher price-earnings ratios experienced a larger negative shock during the outbreak. In the negative-CAR subsample, return on assets (ROA) had a significant and negative impact. ROA to some extent reflects a firm’s ability to generate profits, and the latter is negatively correlated with the volatility of return on investment [63]. At the early stage of the outbreak, economic activities came to a standstill and firms saw a sudden cutoff in revenue. Therefore, more profitable firms were more affected by the pandemic, and their share price reacted more forcefully and negatively to the outbreak.

The previous analysis covers all firms trading on the main boards of SSE and SZSE, as well as the ChiNext startup board. However, the two types of markets impose different listing requirements and standards. Specifically, to be listed on the main boards, a firm has to make continuous profits in the 3 years prior to the offering, have a total share capital of at least RMB 50 million, and it must be relatively mature in business operation and corporate governance. In comparison, ChiNext impose much less stringent listing conditions. On this account, we estimate the coefficients for two subsamples, one for firms trading on the SSE and SZSE main board markets, the other for firm on ChiNext.

Regression results for the main board subsample are displayed in the first part of the Table 7 (Columns 1–3), from which, we can see that the effect of debt-to-asset ratio on CAR was negative but failed to reach significance level, whereas cash flow had a positive and significant impact on CAR. The second part (Columns 4–6) reports regression coefficients for the startup board subsample. We find that for firms trading on ChiNext, debt-to-asset ratio affected their CARs significantly and negatively, and the effect was much larger than that for firms listed on the main boards: on average, an increase of 1 standard deviation (0.19) in debt-to-asset ratio leads to 2.83 percentage point decrease in CAR. Likely, ChiNext firms face more debt constraints than their main board counterparts. As a result, the negative effect of the pandemic was more prominent among these firms. Furthermore, we find net cash flow to have a significant and negative impact on CAR. A possible explanation is, for innovative firms, due to the newness of their business models and fragile operations, those previously recording good cash flow performance may react more severely to the shock, as high information asymmetry could lead to extremely high financing cost (Himmelberg and Petersen, 1994) [64]. As a result, ChiNext firms with healthier cash flow before the pandemic turned out to have suffered more during the outbreak.

**Table 7:** Subsample regression results: SSE & SZSE main board and ChiNext.

	SSE & SZSE main board			ChiNext		
	(1)	(2)	(3)	(4)	(5)	(6)
Lev	-0.0418		-0.0420	-0.1490***		-0.1630***
	(-1.60)		(-1.59)	(-4.97)		(-5.68)
Cash		0.0037**	0.0038**		-1.5470***	-1.9323***
		(2.40)	(2.55)		(-4.93)	(-5.59)
Size	-0.0003	-0.0023	-0.0001	0.0331***	0.0239**	0.0292***
	(-0.12)	(-0.72)	(-0.03)	(3.38)	(2.74)	(3.15)
PE	-0.0026	-0.0013	-0.0026	-0.0086*	-0.0053	-0.0069
	(-0.52)	(-0.24)	(-0.51)	(-1.91)	(-1.26)	(-1.69)
Inventory	0.0028	0.0022	0.0028	0.0045*	-0.0008	0.0044*
	(1.09)	(0.83)	(1.11)	(2.05)	(-0.22)	(2.11)
ROA	-0.0157	0.0637	-0.0146	-0.2635	-0.0246	-0.2562
	(-0.13)	(0.46)	(-0.12)	(-1.66)	(-0.22)	(-1.78)
TobinQ	0.0054	0.0054	0.0053	-0.0024	-0.0004	-0.0021
	(1.49)	(1.43)	(1.48)	(-0.67)	(-0.11)	(-0.62)
Industry fixed effect	Y	Y	Y	Y	Y	Y
Obs.	1800	1800	1800	488	488	488

Notes

1) The t values are shown in brackets

2) all results reported in the table above are net of industry fixed effect at 1-digit level; 3) ***, ** and *, indicate significance at the 1%, 5% and 10% level, respectively.

In terms of controlled variables. Table 7 shows that ChiNext firms were less able to withstand risks than main board firms. Innovative firms may have high price-to-earnings ratios often because investors are willing to pay a premium for potentially high earnings growth. However, when the pandemic hit, investor confidence waned as their fragility were exposed. That is why the negative effect of price-to-earnings ratio was more prominent among ChiNext firms. This finding is consistent with findings in past studies [65–68]. Compared with ChiNext firms, net inventory had a positive effect on firms listed on SSE and SSZE main boards. These firms, larger in size and well-established, tend to maintain a certain level of inventory. Faced with the suddenness of the outbreak and the subsequent production shutdowns, firms with sizable inventory were able to sustain product supply at least for a while, which had positive impact on their stock prices.

In addition, as first-quarter data suggests, the manufacturing sector took a huge hit from the pandemic, leading to a 10.2% decline in its value added [69]. It would be reasonable to postulate that industries featuring high density of people and frequent interactions with the public, such as accommodation and catering, and commercial and leasing services, were likely more affected by the pandemic. Therefore, we carry out regression analysis for two subsamples: the manufacturing subsample, and the service subsample consisting of firms from the accommodation and catering industry and the commercial and leasing services industry. The estimated coefficients are displayed in Table 8.

**Table 8:** Subsample regression results: Manufacturing subsample and service subsample.

	Manufacturing			Accommodation and catering & commercial and leasing services		
	(1)	(2)	(3)	(4)	(5)	(6)
Lev	-0.0579**		-0.0576**	-0.0866		-0.0866
	(-2.28)		(-2.27)	(-1.53)		(-1.53)
Cash		0.0985	0.0834		-0.7169***	-0.7167***
		(1.13)	(0.97)		(-2.83)	(-2.81)
Size	0.0181***	0.0172***	0.0187***	0.0263***	0.0222**	0.0303***
	(2.77)	(2.61)	(2.84)	(2.84)	(2.34)	(3.38)
PE	-0.0102	-0.0092	-0.0100	0.0080	0.0103	0.0110
	(-1.58)	(-1.44)	(-1.55)	(0.85)	(1.19)	(1.17)
Inventory	-0.0028	-0.0054	-0.0028	-0.0019	-0.0041	-0.0036
	(-0.51)	(-1.01)	(-0.51)	(-0.64)	(-1.34)	(-1.17)
ROA	-0.1923	-0.1138	-0.1921	0.4686	0.6349**	0.4847
	(-1.44)	(-0.88)	(-1.43)	(1.46)	(2.40)	(1.61)
TobinQ	0.0007	0.0010	0.0006	-0.0351***	-0.0305***	-0.0345***
	(0.15)	(0.21)	(0.13)	(-2.87)	(-2.96)	(-2.91)
Obs.	1476	1476	1476	39	39	39

Notes

1) The t values are shown in brackets

2) ***, ** and *, indicate significance at the 1%, 5% and 10% level, respectively.

Part 1 of Table 8 (Columns 1–3) corresponds to the manufacturing subsample, Part 2 (Columns 4–6) corresponds to the service subsample (accommodation and catering & commercial and leasing services). The results show that debt-to-asset ratio had a negative and significant impact on CAR: on average, CAR falls by 1.1 percentage points when debt-to-asset ratio rises by 1 standard deviation (0.19); whereas in the service sector, debt-to-asset ratio had no significance. As for cash flow, it’s impact on CAR was insignificant in the manufacturing sector, but in the service sector, the effect was significantly negative. This is likely because compared with manufacturing, in more severely hit services industries, all firms experienced a massive, homogenous shock from the pandemic and were confronted with broad-based operational challenges, even shutdowns; firms in a better cash position before the crisis saw more fluctuations in their cash flow performance, and their share prices thus suffered more damage under the shock of the pandemic.

## 6. Conclusions

By adopting GARCH-based event study, this paper analyzes the impact of the COVID-19 pandemic on the stock performance of firms listed on SSE, SZSE and ChiNext as a whole and cross industries. Our results show that the pandemic had an overall negative impact on the market-wide. However, sizable heterogeneity exists among different industries: The negative impact was especially prominent for contact-intensive sectors such as tourism, retail and catering. While sectors such as healthcare and telecommunication experienced positive performance. Massive demand for masks, antiviral drugs and other related products drove the production and investment in the healthcare sector. New digital business models and working style such as fresh produce e-commerce, telemedicine, online education and remote working expanded rapidly expansion, which boosted stock market performance of the telecommunication operations and courier services sectors.

More importantly, this paper moves on to uncover the micro mechanism behind the heterogeneity of pandemic shock from the perspective of firms’ financial position, focusing particularly on how debt level and pre-pandemic net cash flow affected firms’ stock market performance amid the outbreak. Our results show that firms with higher debt level suffered more due to the pandemic, whereas those with more net cash flow had displayed higher resilience against the blow of the pandemic. This paper then examines whether firms listed on the startup board and those on the main boards reacted differently to the pandemic shock. It finds that as compared with well-established firms trading on the main boards, firms listed on ChiNext are typically small- and medium-sized innovative firms with higher debt-to-asset ratios, which make them more vulnerable to the negative impact of the pandemic. Besides, due to the newness of their business models and fragile operations, those previously recording good cash flow performance have reacted more severely to the shock. This paper further looks at the firm performance in sectors more severely hit by the pandemic such as the service sector, and finds that all firms experienced a massive, homogeneous shock from the pandemic and were confronted with broad-based operational challenges, even complete shutdowns. Firms in a better cash position before the crisis saw more fluctuations in their cash flow performance, and their share prices thus suffered more damage under the shock of the pandemic, thus exhibiting a pattern distinctly different from that of the whole sample.

Our findings suggest that although the impact of the pandemic was negative in general, new drivers of growth, represented by the healthcare sectors as well as digital economy emerged against pandemic headwinds. We believe Chinese economy will see notable heterogeneity in its recovery with the pandemic effectively under control. Certain industries related digital economy and healthcare sectors may even usher in a window of opportunity. This study proves that investors were able to hedge against risks and even gain profits by shifting investment strategy towards firms in the healthcare sector and those with high degree of digitalization during pandemic period.

Furthermore, our findings on micro mechanism are benefit for policy makers to prevent and mitigate systemic financial risk and better facilitate socio-economic recovery. Government should adopt policies to address pandemic-induced cash flow challenges faced by firms and lower their operational costs. For firms carrying big debt loads and severely hit by the pandemic, a variety of measures should be employed to ensure sustained cash flow, such as employment support and wage subsidies, policy-driven loan extension, funds offering bridging loans, etc. For severely impacted service sector, such as such as catering, accommodation and tourism, small and micro business tenants should be allowed reduction or waiver of rent for a period.

As the global epidemic continues to spread, many countries have experienced recurring outbreaks due to local customs, change in climate, and the emergence of new virus strains. Therefore, the implications drawn from this paper in terms of mitigating systemic financial risk and shifting investor strategy could provide a reference for other economies as they respond to pandemic resurgences.
